# The Adequacy of Anesthesia Guidance for Vitreoretinal Surgeries with Preemptive Paracetamol/Metamizole

**DOI:** 10.3390/ph17010129

**Published:** 2024-01-18

**Authors:** Michał Jan Stasiowski, Anita Lyssek-Boroń, Nikola Zmarzły, Kaja Marczak, Beniamin Oskar Grabarek

**Affiliations:** 1 Chair and Department of Emergency Medicine, Faculty of Medical Sciences in Zabrze, Medical University of Silesia, 40-055 Katowice, Poland; 2 Department of Anaesthesiology and Intensive Care, 5th Regional Hospital, Trauma Centre, 41-200 Sosnowiec, Poland; kaja.marczak.wss5@gmail.com; 3 Department of Ophthalmology with Paediatric Unit, 5th Regional Hospital, Trauma Centre, 41-200 Sosnowiec, Poland; anitaboron3@gmail.com; 4 Department of Ophthalmology, Faculty of Medicine, Academy of Silesia, 40-055 Katowice, Poland; 5 Collegium Medicum, WSB University, 41-300 Dabrowa Gornicza, Poland; nikola.zmarzly@gmail.com (N.Z.); bgrabarek7@gmail.com (B.O.G.)

**Keywords:** vitreoretinal surgery, general anesthesia, paracetamol, metamizole, surgical pleth index, Adequacy of Anesthesia, numeric rating scale

## Abstract

Despite the possibility of postoperative pain occurrence, in some patients, vitreoretinal surgeries (VRSs) require performance of general anesthesia (GA). The administration of intraoperative intravenous rescue opioid analgesics (IROA) during GA constitutes a risk of perioperative adverse events. The Adequacy of Anesthesia (AoA) concept consists of an entropy electroencephalogram to guide the depth of GA and surgical pleth index (SPI) to optimize the titration of IROA. Preemptive analgesia (PA) using cyclooxygenase-3 (COX-3) inhibitors is added to GA to minimize the demand for IROA and reduce postoperative pain. The current analysis evaluated the advantage of PA using COX-3 inhibitors added to GA with AoA-guided administration of IROA on the rate of postoperative pain and hemodynamic stability in patients undergoing VRS. A total of 165 patients undergoing VRS were randomly allocated to receive either GA with AoA-guided IROA administration with intravenous paracetamol/metamizole or with preemptive paracetamol or metamizole. Preemptive paracetamol resulted in a reduction in the IROA requirement; both preemptive metamizole/paracetamol resulted in a reduced rate of postoperative pain as compared to metamizole alone. We recommend using intraoperative AOA-guided IROA administration during VRS to ensure hemodynamic stability alongside PA using both paracetamol/metamizole to reduce postoperative pain.

## 1. Introduction

Ophthalmic surgery has witnessed a major transformation from performance of vitreoretinal surgeries (VRSs) traditionally under general anesthesia (GA) in the 1990s to utility of a wide variety of regional anesthesia (RA) techniques in the present century [1]. Nevertheless, there are contraindications to perform RA alone, including antiplatelet therapy, predicted impaired cooperation with the operator during long-lasting procedures due to neurological deficits, and danger of respiratory disorders during pars plana vitrectomy (PPV) under RA with monitored anesthesia care in elderly patients, which still imposes the necessity of VRS performance under GA [2,3]. Although attempts have been made to perform VRS under RA in patients using oral anticoagulants [4], it entails the administration of intraoperative rescue opioid analgesia (IROA) in the case of an increased heart rate and blood pressure, which are signs of inadequate analgesia. The utility of IROA has been identified as an independent risk factor for postoperative nausea and vomiting (PONV) in the first 48 h after VRS [4], and therefore RA techniques are employed to reduce the dose of IROA. The addition of different preoperative, preventive RA techniques to GA in patients undergoing VRS also reduces the IROA dose [5,6], which in turn decreases the rate of PONV, leading to efficient postoperative analgesia and diminishing the rate of the oculocardiac reflex (OCR), although it was not completely eliminated [7].

Metamizole and paracetamol (acetaminophen) are the two most widely used non-opioid analgesics, possessing both a central (inhibition of cyclooxygenase-3, COX-3) and a peripheral mechanism of action [8,9,10,11,12], which have proven their analgesic efficacy in the treatment of postoperative pain with a single dose administered as preemptive analgesia [13,14,15,16,17,18], in reducing the incidence of PONV [13,14] and a quite good safety profile [11,19,20,21] compared to standard nonsteroidal anti-inflammatory drugs (NSAIDs), in terms of safety for the upper intestinal tract and kidneys in patients with an increased risk of stomach or renal problems or other contraindications for standard NSAIDs [22,23].

Surgical painful (nociceptive) afferent stimulation in the operation field during GA in the case of insufficient intraoperative analgesia leads to the stress hormone release, which triggers an increase in heart rate and arterial blood pressure, whereas the administration of IROA attenuates this effect. On the one hand, an inadequate dosing may be responsible for intolerable postoperative pain perception (IPPP) [24] in the mechanism of central sensitization [25]; on the other hand, an excessive dosage may lead to hemodynamic instability with dangerous bradycardia and hypotension as well as an increased requirement for intravenous fluids.

Hence, appropriate titration of IROA constitutes a major challenge in response to observed fluctuations of abovementioned hemodynamic parameters accompanied by anesthesiologic intuition, in view of the fact that insufficient intraoperative analgesia may not necessarily be reflected in tachycardia and hypertension as volatile anesthetics tend to blunt the hemodynamic response to nociceptive stimulation [26], especially in the population of diabetics and the elderly.

Intraoperative monitoring of analgesia efficacy is crucial and is therefore gaining increasing popularity in daily anesthesiological practice as control of the intraoperative efficacy of IROA administration during GA, reflecting the nociception/antinociception balance [27], was proven to be more effective compared to IROA administration, based on observation of hemodynamic changes in reaction to painful stimuli intraoperatively [28,29].

The Adequacy of Anesthesia (AoA) concept is based on monitoring the depth of GA detected from a forehead sensor using an entropy electroencephalogram (Response Entropy, RE; State Entropy, SE) and the surgical pleth index (SPI) derived from a finger photoplethysmography signal, both of which do not require complex preoperative preparations [30].

Observance of the SE value within the range of 40–60 as a result of proper administration of the hypnotic GA component, reflecting the proper suppression of the limbic system, alongside observance of the increase in the SPI value on the monitor (0—no painful stimulation, 100—maximum painful stimulation) after a painful stimulus and returning to the baseline level after the IROA bolus, makes the monitoring with AoA guidance easy [31]. The reliability of variations in SPI values in response to nociceptive stimulation was proven to correlate with serum opioid concentration [32] and reduce the requirement for total cumulative IROA during GA [33]. The employment of AoA guidance for IROA titration also proved its utility in decreasing postoperative pain perception expressed on the numerical rating scale (NRS) compared to standard practice in patients undergoing lumbar discectomy [34].

To the best of our knowledge, so far, no study has been carried out in order to investigate the potential effect of combined preemptive analgesia (PA) using two different cyclooxygenase type 3 inhibitors together in patients undergoing VRS under GA with AoA guidance on the incidence of postoperative pain and hemodynamic stability. Therefore, the aim of the current study was to assess the utility of AoA guidance of GA in patients undergoing VRS with combined PA using both paracetamol and metamizole or one of them.

## 2. Results

**Figure 1:**
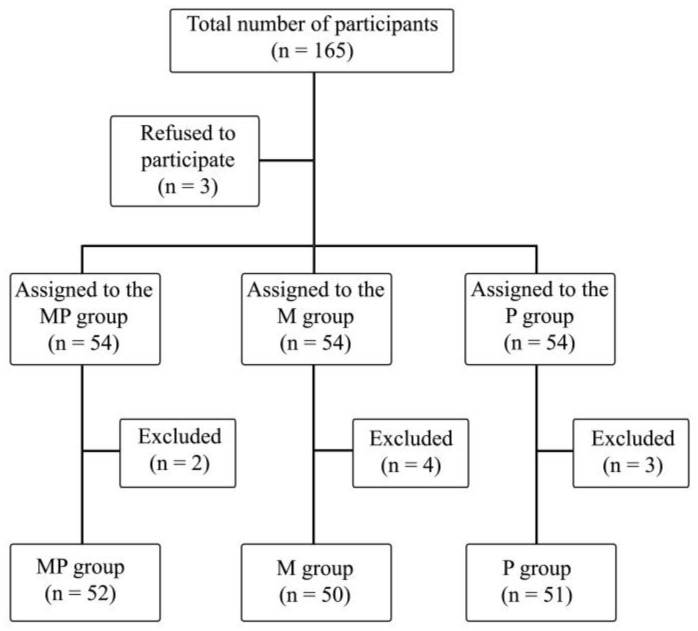
Randomization graph. PM—paracetamol/metamizole group; M—metamizole group; P—paracetamol group.

The study included 165 patients, of which 9 patients were excluded due to technical problems with intraoperative SPI measurement, postoperative arousal leading to inability of observation during stage 5, or inability of declaring postoperative pain perception. A total of 153 patients’ data were ultimately analyzed (Figure 1).

Table 1 shows the anthropometric data of the patients. Significant differences were noted for height, weight, and BMI.

We analyzed the frequency of surgical maneuvers during the procedure (Table 2) among the groups to ensure the homogeneity of the groups according to intraoperative nociception received by patients, which could induce the severity of postoperative pain. There was a statistically significant difference in the case of gas–liquid exchange, which occurred less frequently in the PM group compared to the M group. Significant differences were also noted for silicon oil injection between the PM and P groups.

**Figure 2:**
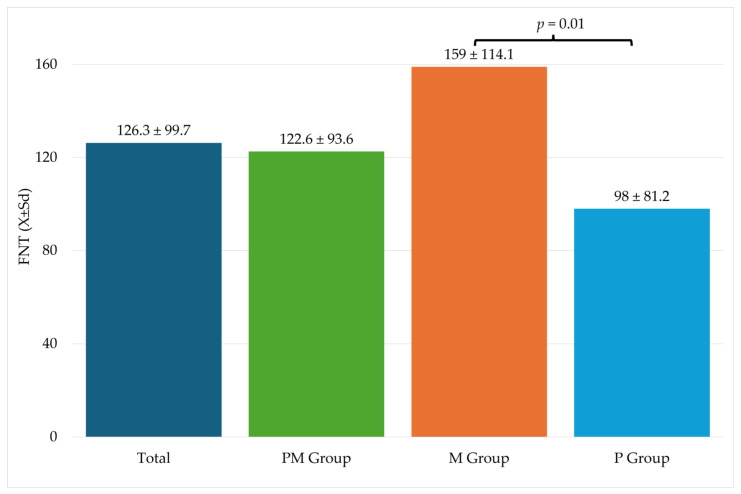
Fentanyl use during surgery. FNT—fentanyl; M—metamizole group; P—paracetamol group; Sd—standard deviation.

Table 3 shows information related to the procedure. Significantly higher FNT values were recorded in the M group compared to the P group (Figure 2).

**Figure 3:**
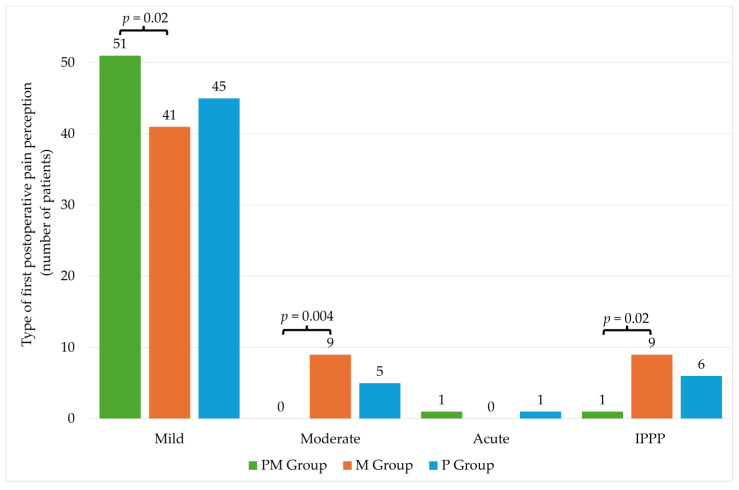
Type of first postoperative pain perception. PM—paracetamol/metamizole group; M—metamizole group; P—paracetamol group; IPPP—intolerable postoperative pain perception.

There were no statistically significant differences in individual groups in the level of pain intensity on the NRS scale (Table 4). The significantly higher percentage of patients with mild pain was observed in the PM group compared to the M group. The opposite situation was observed in the case of moderate pain, where a significantly higher percentage occurred in the M group. No significant differences were noted for acute pain. In the case of IPPP, there were differences between PM and M groups (Figure 3).

Table 5 shows hemodynamic changes during individual stages of anesthesia. Significant differences were noted only during stage 4 between groups M and P in the case of mean SAP and mean MAP. These values were higher in the P group.

Table 6 shows hemodynamic fluctuations. In stage 3, significantly higher levels of min SAP and min MAP were recorded in the PM group compared to the M group. In stage 4, max HR was significantly higher in the PM group compared to the M group. Significant differences were also noted for min SAP, which achieved the highest value in the P group. Moreover, in the case of min DAP, the highest value was also recorded in the P group. In stage 5, significant differences were recorded only in the case of max SPI. Its value was significantly higher in the PM group compared to the M group.

## 3. Discussion

The current study analysis covering the utility of PA using paracetamol and metamizole in patients undergoing VRS revealed the incidence of IPPP in 16 out of 153 patients (10.46%) despite the allocation to groups. In a group of patients receiving combined PA using metamizole and paracetamol, only 1 out of 53 patients (1.88%) experienced postoperative pain of a type other than mild, whereas the utility of preemptive metamizole resulted in an incidence of IPPP in 9 out of 50 patients (18%) and that of preemptive paracetamol resulted in an IPPP incidence in 6 out of 50 patients (12%), which appeared to be statistically significant (*p* < 0.05). Only in one patient from the PM group was another postoperative pain type other than mild observed, which seems to be quite an achievement in view of the fact that the majority of patients received noxious surgical maneuvers like endolaser treatment and indentation, which we had already proved in our other study from this field [35].

The impact of the burgeoning geriatric and diabetic population has increased requirements for VRS, whereas concomitant diseases, anti-platelet therapy, and risk of globe perforation due to impaired cooperation with the operator during a long-lasting procedure may possibly exclude performance of VRS under RA with monitored anesthesia care. Therefore, the necessity of employment of the GA regimen entailing the use of IROA encourages us to seek PA modalities, which were reported to diminish the requirement for IROA and as a result reduce the presence of perioperative adverse events like postoperative pain, PONV, and OCR, similarly to RA techniques added to GA as PA.

In patients with a contraindication for NSAIDs, pain management is challenging; therefore, metamizole and paracetamol are extensively used as an alternative to classic NSAIDs and opioids in postoperative pain management [22]. As part of a multimodal concept after minor-to-intermediate surgery, both paracetamol and metamizole, when administered intravenously, were proven to possess equivalent analgesic efficacy, being widely employed to achieve satisfactory improvement in postoperative pain therapy [23,36,37], although there are conflicting data in terms of the influence of their use on the IROA sparring effect and reduction in IROA-dosing-related adverse events, like nausea and vomiting, mainly due to a difference in anesthetic regimens [36,38,39].

Paracetamol is widely used in the management of acute and chronic pain [19], and its analgesic efficacy as a preemptive analgesia has already been well-recognized in patients undergoing lumbar discectomy [40], total abdominal hysterectomy [41], lower limb surgery [42], and cholecystectomy [43], whereas metamizole was successfully used in the management of pain after laparoscopic operations [44].

In the course of the current study, no adverse events connected with metamizole or paracetamol administration were observed; nevertheless, their utility is not free from potential side effects [45,46]. Metamizole is a controversial drug reported to incidentally induce agranulocytosis [23,47,48,49], anaphylaxis [50], Kounis syndrome (coincidental occurrence of allergic reaction and acute coronary syndrome secondary to vasospasm) [51]. Paracetamol, on the other hand, can have side effects such as tachycardia, hypertension, hepatotoxicity, and nephrotoxicity [52], and its use for pain control has been discussed in some studies [53].

Despite potential drawbacks, their analgesic efficacy was also utilized for the reduction in postoperative pain perception in ophthalmic surgeries, as pain intensity after a vitrectomy with scleral buckling has been reported to be the most severe [54]. De Araújo et al. used 1 g of metamizole in an intravenous infusion 40 min before panretinal photocoagulation and recorded significant reduction in the pain perception associated with the procedure as compared to the placebo control group [55]. Landwehr et al. compared analgesic potency of intravenous paracetamol and intravenous metamizole both in a dose of 1 g and proved their similar efficacy for postoperative analgesia after retinal surgery compared to the control group [56]. Interestingly, metamizole was proven to also act via substance P and the cannabinoid system [57,58] with the involvement of a possible higher reduction in IROA compared to that observed in our previous study [59] and IROA-related postoperative adverse events compared to that in our additional report [60]. In this study, we used 2.5 g of metamizole as PA and observed the incidence of IPPP in 18% of our patients in the metamizole group, whereas in our previous study, we used 1 g of metamizole and observed IPPP in 22.9% of patients [59]. Increasing the dose of metamizole used as PA to 2.5 g in a single dose with the subsequent reduction in its postoperative dosing to a 5 g daily cumulative dose did not lead to proportional reduction in IPPP and we find it interesting but questionable also due to the fact that the IROA dose in this study was 159 ± 114.1 mcg in the metamizole group compared to 165.7 ± 116.8 mcg in the previous study [59].

In the case of paracetamol, Sadrolsadat et al. investigated its influence when administered either as PA preoperatively or upon emergence from GA compared to the control group in patients undergoing PPV. They found pain scores to be lower in both the paracetamol groups, compared with the control at recovery [61].

Numerous abovementioned studies prove that the addition of intravenous PA to GA may reduce the demand for IROA administration, which was proven in the current study in the case of paracetamol contrary to metamizole and combined PA using both paracetamol and metamizole. Interestingly, a statistically significant difference of demand for IROA using FNT between groups M and P was observed (likewise in our previous study in a smaller group of patients) [59]. Interestingly, an inexplicably higher demand for IROA was noted in the M group compared to the PM group, although not statistically significant, similar to that between the M group and control group without PA in the previous study [59]. In a study performed by Ledowski et al. [62], the variations of the SPI value after surgical stimulation and redressing the value down to a baseline level after a bolus of rescue FNT enabled them to monitor the efficacy of IROA titration; therefore, it was also assumed that AoA guidance of IROA administration in our study helped monitor the effectiveness of every single rescue bolus of FNT in the case of insufficient analgesia produced by intravenous PA.

Employment of similar anesthetic modality in the current study analysis also revealed that IROA cannot be eliminated completely, even if the use of SPI in guidance of IROA administration in the case of intraoperative afferent nociceptive stimulation due to incomplete efficacy of PA using intravenous preemptive analgesia with either paracetamol or metamizole or combined PA using both of them was employed. Therefore, the use of AoA-guided IROA administration during GA, whenever the patients experienced afferent intraoperative nociceptive stimulation (any incident of intraoperative ∆SPI > 15 compared to SPI value before the start of VRS in stage 2 constituting an indication for ROA administration), was observed to produce more efficient suppression of central sensitization in the case of insufficient intravenous PA using paracetamol or metamizole detected using SPI monitoring.

The employment of AoA guidance of GA for VRS resulted in the achievement of hemodynamic stability, because in the majority of cases, no statistically significant differences were observed between analyzed parameters between studied groups in terms of SAP, MAP, DAP, and HR. In no case was the intraoperative use of vasopressors needed, which for some authors is included in the intraoperative hypotension classification [63]. Several statistically significant differences were of no clinical value as the values of hemodynamic parameters did not meet criteria of hypotension understood as blood pressure values’ difference of more than 30% from the baseline values [64] that might have resulted in multiorgan failure, leading to heart attack or stroke [65] or conversely malignant hypertension with risk of pulmonary edema.

There are several limitations of the current analysis. First of all, the perception of postoperative pain is a subjective phenomenon, which is burdensome in quantification [66]. We did not analyze the rate of postoperative pain after discharge from PACU to the DoL on purpose as the project involved monitoring NRS as well as SPI values in stage 5, whereas patients’ arousal (changing position in bed, cough, etc.) markedly interferes in SPI monitoring [67]; therefore, such comparison has no clinical value [68]. The rate of incidence of PONV, OCR, and oculo-emetic reflex will be analyzed separately (likewise in our previous study in this field) [60]. Similarly, intraoperative pain perception during certain stages of VRS will also be separately analyzed. Second, 9 of 165 patients were excluded from the final analysis due to technical problems with intraoperative SPI measurement, postoperative arousal leading to inability of observation during stage 5, or inability of declaring postoperative pain perception, which may have proportionally influenced the final results. Finally, we intentionally did not study the groups without AoA guidance, as the current study design is based on the previous study findings with a different PA regimen under AoA guidance compared to AoA guidance of IROA administration alone, with the intention of investigating the utility of combined paracetamol/metamizole compared to their separate use under AoA guidance. An anesthesia regimen including monitoring the depth of anesthesia, regardless of the technique used, and monitoring the nociception/antinociception balance, regardless of whether SPI, antinociception index, or pupillometry of the nociception level was applied, is becoming a global standard; therefore, the administration of IROA based on observing changes in hemodynamic values alongside anesthesiological intuition may no longer be justified in light of the availability of current medical technology and thus raises ethical concerns.

## 4. Materials and Methods

### 4.1. Patients

Patients who were scheduled for elective VRS in the Department of Ophthalmology of the 5th Regional Hospital in Sosnowiec, Poland, and met the inclusion criteria were asked to participate in the study. No previous surgical procedures, no history of increased intraocular pressure, and no treatment with antiglaucoma drugs were the inclusion criteria. Exclusion criteria involved factors affecting the anatomy or function of the posterior segment of the eye that predispose to glaucoma, including penetrating ocular trauma, uveitis, scleral buckling, retinal vein occlusion. One hundred and sixty-five patients with American Society of Anaesthesiologists (ASA) score I–III were enrolled after obtaining written informed consent. Then, randomization was performed by opening sealed envelopes. In compliance with the Helsinki Declaration, ethical approval for this study (KNW/0022/KB1/122/17) was provided by the Ethical Committee of Medical University of Silesia on 5 December 2017 (Chairman Ph.D., MD Bogusław Okopień). The project was registered in the Clinical Trial Registry (Silesian MUKOAiIT7, NCT03389243). Data were collected between 15 January 2018 and 16 December 2022.

Exclusion criteria were pregnancy, drug or alcohol abuse, history of neurological disease or neurosurgical operation that would impair entropy electroencephalogram monitoring and history of pulmonary disease, history of allergy to or abuse of metamizole or paracetamol, acute or chronic pain, signs predicting difficult laryngeal mask placement, cardiac arrhythmia in ECG that might impair SPI monitoring.

Patients were randomly allocated into three groups. In group M, patients received GA anesthesia in addition to PA using a single dose of 2.5 g of metamizole (Pyralgin at 0.5 g/mL, 5 mL solution; Polpharma S.A., Gdansk, Poland) in 100 milliliters of a saline solution 30 min before arrival to the operating room. In group P, patients received GA anesthesia in addition to PA using a single dose of 1 g of acetaminophen (Paracetamol Kabi at 10 mg/mL, solution of 100 mL; Fresenius Kabi, Błonie, Poland) in 100 milliliters of a saline solution 30 min before arrival to the operating room. In group PM, patients received GA in addition to combined abovementioned PA techniques in the P and M group, according to the latest change in the guidelines for postoperative pain management [69].

The day before VRS during the preoperative visit, patients were informed about the possibility of postoperative pain. They were trained on the use of the Numeric Pain Rating Scale (NRS) regarding how to report pain. On a scale from 0 to 10, 0 indicated no pain and 10 indicated the worst pain imaginable.

### 4.2. Anesthesia Technique

Regardless of group allocation, patients were kept fasted for at least 12 h and before the induction of anesthesia started, on the day of surgery, all patients received medication in a dose of 3.75–7.5 mg/kg of midazolam (Dormicum, Roche, Warsaw, Poland) in accordance with age and body weight [70]. Directly before surgery, the patients were preoxygenated for approximately 5 min with 100% oxygen and intravenously 10 mL/kg body weight of an Optylite solution was infused. The preoxygenation time was intended to ensure patient safety and avoid any potential risk. Anesthesia was induced intravenously with fentanyl at 1 mcg/kg body weight, and Propofol (Propofol 1% Fresenius, Cairo, Egypt) at a single dose of 2.5 mg/kg body weight. After loss of consciousness, the patients in all groups were paralyzed with a standard intravenous dose of 0.6 mg/kg rocuronium (Esmeron, Fresenius, Błonie, Poland) and after 45 s, the laryngeal mask (LMA) was placed. CO_2_ was maintained at 35–37 mmHg etCO_2_. After LMA placement, before operation started, sevoflurane concentration was maintained at the level around 40–45 State Entropy.

Throughout anesthesia induction and surgery, standard monitoring procedures were utilized and close attention was paid to vital parameters such as the non-invasive arterial pressure (NIBP), heart rate (HR), standard electrocardiography (ECG), arterial blood saturation (SaO_2_), fraction of inspired oxygen in the gas mixture (FiO_2_), fraction of inspired sevoflurane (FiAA), fraction of expired sevoflurane (FeAA), exhaled carbon dioxide concentration (etCO_2_), minimal alveolar concentration of sevoflurane (MAC). The depth of anesthesia was monitored with the entropy electroencephalogram (State and Response Entropy), intraoperative analgesia was guided with the surgical pleth index (SPI), and muscle relaxation was maintained using NMT monitoring (Carescape B650, GE, Helsinki, Finland).

#### 4.2.1. Stage 1

On admission to the operating theater, the sensor of the entropy electroencephalogram (RE, SE) on the patients’ forehead, the pulse oximeter (for SPI measurement) on the contralateral finger to venous access and NIBP cuff on the right arm, and standard ECG on the patients’ back were placed according to the manufacturer’s suggestions, and the first values were recorded.

#### 4.2.2. Stage 2

In order to calculate the mean SPI value during stage 2, SPI values were taken into account starting from 5 min after laryngeal mask placement to the beginning of the sterilization of the orbit to allow the calibration of the SPI sensor.

#### 4.2.3. Stage 3—Intraoperative

The SPI score was monitored online and recorded with a sampling frequency of 1 min. When the SPI value reached ∆SPI > 15 points above the mean SPI value in stage 2, a rescue dose of 1 mcg/kg body weight of FNT was administered intravenously every 5 min until the SPI value decreased to the value of mean SPI recorded in stage 2. The time duration of VRS was counted from the speculum installation to the speculum removal.

We assumed that the initial dose of FNT of 1 mcg per kilogram of body weight would produce sufficient analgesia before the installation of the speculum. In addition, in 2013, Gruenewald et al. [27] proposed ∆SPI > 10 or an absolute SPI value > 50 as a predictor of inadequate analgesia. In other studies, only absolute value ∆SPI > 50 became an indication for rescue analgesia [71]. In the methodology of the current study, a compromising protocol of ∆SPI > 15, compared to the calculated baseline during stage 2, lasting at least one minute as an indicator for rescue analgesia was adopted to avoid a potentially hazardous overdosage of FNT as a result of the potential miscalculation of the SPI score due to its variations.

VRSs were performed by the same ophthalmic surgeon (A.L.-B.) with over 10 years of experience in VRS, performing over 400 vitreoretinal procedures a year. Eye globe preparation included three or four 23-gauge ports. Initial core vitrectomy was followed by peripheral vitreous removal with scleral indentation. The removal of epiretinal membranes and/or internal limiting membrane peeling was the next surgical step. If needed, 4 sorts of tamponades were applied: temporary heavy perfluorocarbon liquids, air, SF6, or silicon oil. All retinal brakes or degenerations were treated with laser photocoagulation.

The incidence of intraoperative OCR was recorded, which is typically identified in the case of a rapid decrease in heart rate by 20% from the baseline during ocular manipulations. In the case of the presence of OCR, the surgeon was asked to stop stimulation in the surgical field, and intravenous atropine was administered intravenously in a single dose of 0.5 milligrams when the hemodynamic manifestation of OCR did not withdraw.

#### 4.2.4. Stage 4—Emergence from GA

All the patients were monitored further (SPI, HR, SAP, MAP, DAP, SaO_2_) by the anesthesiological team performing GA.

#### 4.2.5. Stage 5—Postoperative

In the post-anesthesia care unit (PACU), all the patients were monitored further (SPI, HR, SAP, MAP, DAP, SaO_2_) by the anesthesiological team blinded to the patients’ group allocation. Along with postoperative hemodynamic parameters, the presence of adverse effects such as nausea and vomiting (PONV), level of sedation, and allergic reactions was monitored for each patient at the same time as pain assessment for 24 h. In the case of PONV, ondansetron (Ondansetron Accord, Accord Healthcare Limited, Harrow, UK) in a single intravenous dose of 4 mg was administered intravenously. The Optylite solution at 5 milliliters/kg body weight was infused in the case of MAP < 65 mmHg. The patients received oxygen at 3 L/min via a nasal cannula. The patients were questioned in terms of their perception of pain intensity using the Numeric Pain Rating Scale (NRS) ranging from 0 (no pain) to 10 (maximum pain) every 10 min. In the case of pain perception NRS > 3, a standard dose of NSAID to meet each patient’s individual needs, like dexketoprofen (Dexak, Berlin-Chemie Menarini, Berlin, Germany) or ibuprofen (Ibuprofen B.Braun Melsunngen, Germany), alongside paracetamol or metamizole, was administered according to contemporary guidelines of acute pain treatment issued by the Polish Society of Anaesthesiologists [24]. SPI values were monitored online and mean SPI values were recorded with a sampling time of 1 min (trends in software provided by producer). NRS and SPI values were recorded for acute pain (NRS 7–10), average pain (NRS 4–6), and mild pain (NRS 0–3) perception intervals. The patients were observed and monitored in the PACU for at least 30 min until transfer from the PACU to Department of Ophthalmology. The monitoring and data recording were then ceased with the exception of the incidence of PONV, which was postoperatively recorded during the first 24 h and each time anti-emetic ondansetron (Ondansetron Accord at 2 mg/mL, 2 mL solution, Accord Healthcare Limited, Great Britain) was administered intravenously in a dose of 4 milligrams.

### 4.3. Statistical Analysis

Statistical calculations were performed using STATISTICA 13.3 (StatSoft, Kraków, Poland). Quantitative data were characterized using the mean and standard deviation as well as the median and interquartile range. Qualitative data were presented using percentages. Normality of distribution was verified with the Shapiro–Wilk test. Due to the lack of normal distribution, a quantitative data analysis was performed using the Kruskal–Wallis test and Dunn’s post hoc test. The chi-square test was used to analyze qualitative data. Statistical significance was set at the level *p* < 0.05.

#### Sample Size Calculation

We took into account the time of onset results of a previous study with 35 patients per group. The aim was to detect a significant difference in the time of onset of anesthesia among the paracetamol, metamizole, and paracetamol- and metamizole-alone groups. The calculation was based on a one-way ANOVA test conducted with α = 0.05 and a power of 80%, and the sample size was calculated as 55 patients per group.

## 5. Conclusions

In conclusion, contrary to numerous studies proving efficacy of PA using COX-3 inhibitors, our analysis proved an advantage of the intravenous infusion of combined PA using paracetamol and metamizole before VRS under AoA guidance in terms of incidence of postoperative pain perception. In addition, preemptive paracetamol administration diminished the requirement for intravenous FNT.

Utility of preemptive analgesia using both paracetamol and metamizole seems to be justified in terms of the observed tendency for improved postoperative analgesia, whereas paracetamol alone tends to reduce intraoperative demand for IROA.

## Figures and Tables

**Table 1:** Anthropometric data in studied groups of patients.

Metrics		Total	PM Group	M Group	P Group	*p*-Value
		N = 153 (100%)	N = 52 (34%)	N = 50 (32.7%)	N = 51 (33.3%)	
Age X ± Sd Me (IQR)	years	63.8 ± 11.7	64.4 ± 11	62.6 ± 12.3	64.6 ± 11.6	0.7
		65 (12)	67 (11.5)	64 (14)	66 (9)	NS
Gender, N (%)	female	82 (53.6)	23 (44.2)	24 (48)	35 (68.6)	0.05
	male	71 (46.4)	29 (55.8)	26 (52)	16 (31.4)	NS
Anthropometric data		Total	PM Group	M Group	P Group	*p*-Value
Height X ± Sd Me (IQR)	cm	167.1 ± 9.8	169.5 ± 9	168.3 ± 9.4	164 ± 8.3	PM vs. P, *p* = 0.015
		165 (15)	167 (12)	170 (15)	164 (11)	M vs. P, *p* = 0.047
Weight X ± Sd Me (IQR)	kg	77.2 ± 15.4	85 ± 15.6	74.6 ± 13.9	74 ± 14.8	PM vs. M, *p* = 0.009
		77 (21)	82 (27)	74.5 (19)	72.5 (21.5)	PM vs. P, *p* = 0.004
BMI X ± Sd Me (IQR)	kg/m^2^	27.5 ± 5.5	29.6 ± 5	26.3 ± 4.2	27.1 ± 6.7	PM vs. M, *p* = 0.008
		26.6 (6.7)	27.8 (5.9)	25.6 (4)	27.6 (7.4)	
BMI N (%)	underweight	3 (2)	0 (0)	1 (2)	2 (4)	0.21
	norm	45 (29.4)	8 (15.4)	20 (40)	17 (33.3)	
	overweight	49 (32)	14 (26.9)	20 (40)	15 (29.4)	NS
	obesity	39 (25.5)	15 (28.8)	9 (18)	15 (29.4)	

PM—paracetamol/metamizole group; M—metamizole group; P—paracetamol group; Sd—standard deviation; Me—median; IQR—interquartile range; BMI—body mass index; NS—not significant.

**Table 2:** The frequency of surgical maneuvers (numbers and percentages) during the procedure.

Type of VRS	Total N = 153 (100%)	PM Group N = 52 (34%)	M Group N = 50 (32.7%)	P Group N = 51 (33.3%)	*p*-Value
Pars Plana Vitrectomy	62 (40.5)	23 (44.2)	17 (34)	22 (43.1)	*p* = 0.52 NS
Yes (%)					
Phacovitrectomy	91 (59.5)	29 (55.8)	33 (66)	29 (56.9)	*p* = 0.52 NS
Yes (%)					
The frequency of surgical maneuvers during VRS					
Speculum Adjustment	153 (100)	52 (100)	50 (100)	51 (100)	-
Yes (%)					
Trocars’ Insertion	153 (100)	52 (100)	50 (100)	51 (100)	-
Yes (%)					
Vitrectom Insertion	153 (100)	52 (100)	50 (100)	51 (100)	-
Yes (%)					
Staining Agent Injection	128 (83.7)	46 (88.5)	41 (82)	41 (80.4)	*p* = 0.5 NS
Yes (%)					
Peeling	135 (88.2)	46 (88.5)	45 (90)	44 (86.3)	*p* = 0.8 NS
Yes (%)					
Gas–Liquid Exchange	51 (33.3)	10 (19.2)	23 (46)	18 (35.3)	PM vs. M, *p* = 0.007
Yes (%)					
Endolaser Treatment	120 (78.4)	39 (75)	43 (86)	38 (74.5)	*p* = 0.3 NS
Yes (%)					
Silicon Oil Injection	52 (34)	24 (46.2)	17 (34)	11 (21.6)	PM vs. P, *p* = 0.02
Yes (%)					
Indentation	58 (37.9)	14 (26.9)	18 (36)	26 (51)	*p* = 0.05 NS
Yes (%)					
Subconjunctival Injection	151 (98.7)	50 (96.2)	50 (100)	51 (100)	-
Yes (%)					
Trocars’ Removal	153 (100)	52 (100)	50 (100)	51 (100)	-
Yes (%)					
Speculum Removal	153 (100)	52 (100)	50 (100)	51 (100)	-
Yes (%)					

VRS—vitreoretinal surgery; PM—paracetamol/metamizole group; M—metamizole group; P—paracetamol group; NS—not significant.

**Table 3:** Characteristics of the performed surgery.

Surgery		Total	PM Group	M Group	P Group	*p*-Value
		N = 153 (100%)	N = 52 (34%)	N = 50 (32.7%)	N = 51 (33.3%)	
Time of VRS X ± Sd Me (IQR)	min	47.2 ± 18.9	44.4 ± 19	50 ± 19.4	47.2 ± 18.3	*p* = 0.3 NS
		44 (28)	39 (24)	51 (31)	44 (29)	
FNT X ± Sd Me (IQR)	mcg	126.3 ± 99.7	122.6 ± 93.6	159 ± 114.1	98 ± 81.2	M vs. P, *p* = 0.01
		100 (150)	100 (125)	150 (200)	100 (50)	
Intraoperative fluid therapy X ± Sd Me (IQR)	mL	1042.2 ± 342.6	1090.4 ± 389.4	1069 ± 351.1	958.7 ± 258.5	*p* = 0.5 NS
		1000 (450)	1100 (450)	1000 (450)	1000 (250)	

VRS—vitreoretinal surgery; PM—paracetamol/metamizole group; M—metamizole group; P—paracetamol group; Sd—standard deviation; Me—median; IQR—interquartile range; FNT—fentanyl; NS—not significant.

**Table 4:** Assessment of the occurrence of postoperative pain in the examined groups.

Postoperative Pain		Total	PM Group	M Group	P Group	*p*-Value
		N = 153 (100%)	N = 52 (34%)	N = 50 (32.7%)	N = 51 (33.3%)	
NRS max X ± Sd Me (IQR)	[1 ÷ 10]	1 ± 1.8	0.5 ± 1.4	1.3 ± 2.1	1.1 ± 1.9	0.1 NS
		0 (2)	0 (0)	0 (3)	0 (2)	
Type of first postoperative pain perception N (%)	Mild	137 (90)	51 (98)	41 (82)	45 (88.2)	PM vs. M, *p* = 0.02
	Moderate	14 (9)	0 (0)	9 (18)	5 (9.8)	PM vs. M, *p* = 0.004
	Acute	2 (1)	1 (2)	0 (0)	1 (2)	0.6 NS
	IPPP	16 (10)	1 (2)	9 (18)	6 (11.8)	PM vs. M, *p* = 0.02

PM—paracetamol/metamizole group; M—metamizole group; P—paracetamol group; Sd—standard deviation; Me—median; IQR—interquartile range; NRS—numerical rating scale; IPPP—intolerable postoperative pain perception; NS—not significant.

**Table 5:** Hemodynamic changes in patients with intraoperative pain perception during certain stages of anesthesia.

Parameter X ± Sd Me (IQR)	PM Group	M Group	P Group	*p*-Value
	N = 52 (34%)	N = 50 (32.7%)	N = 51 (33.3%)	
Stage 1—onset				
SAP (mmHg)	155.6 ± 22.5	152 ± 18.5	151.4 ± 19.1	*p* = 0.6
	158 (29)	150.5 (29)	154 (28)	NS
MAP (mmHg)	111.6 ± 13.5	110 ± 11.5	110 ± 11.7	*p* = 0.7
	111 (18.5)	108 (19)	110 (15)	NS
DAP (mmHg)	78.8 ± 11.1	80.4 ± 9.4	78.4 ± 10.3	*p* = 0.6
	78 (17.5)	79.5 (10)	76 (15)	NS
HR (beats/min)	73.5 ± 13	72.1 ± 12.6	73.6 ± 11.2	*p* = 0.8
	72 (19.5)	72.5 (19)	74 (16)	NS
SPI	58.2 ± 16.2	57.4 ± 17.5	57.1 ± 16.8	*p* = 0.8
	58 (23)	59 (21)	54 (27)	NS
Stage 2—between induction and start of VRS				
mean SAP (mmHg)	124.9 ± 30.1	125.2 ± 28.1	131.7 ± 27.1	*p* = 0.2
	117 (39.5)	126.7 (34.5)	131 (43.5)	NS
mean MAP (mmHg)	91.6 ± 19	95 ± 16.1	96.4 ± 17.8	*p* = 0.2
	91.5 (23.8)	96.8 (22.5)	97 (27)	NS
mean DAP (mmHg)	68 ± 13.2	72.7 ± 12.7	71.7 ± 12.7	*p* = 0.1
	68.8 (16)	71.8 (23)	72 (19.5)	NS
mean HR (beats/min)	67.2 ± 10.8	68.1 ± 13.4	68.6 ± 9.9	*p* = 0.5
	64.6 (15.2)	71 (17.7)	67.3 (13.3)	NS
mean SPI	33.3 ± 15.8	34 ± 12.3	40.2 ± 35.2	*p* = 0.2
	29.3 (20.3)	31.7 (12.6)	33 (17.9)	NS
mean SE	41.5 ± 9.3	41.4 ± 10.6	40.4 ± 10.4	*p* = 0.8
	41.2 (12.9)	42.7 (14)	30.4 (17)	NS
Stage 3—VRS				
mean SAP (mmHg)	117.4 ± 23.4	107.7 ± 14.7	111.3 ± 25.3	*p* = 0.2
	112.8 (30.4)	108.1 (23)	105.5 (26.7)	NS
mean MAP (mmHg)	87.6 ± 14.8	82.1 ± 10.6	84.2 ± 15.2	*p* = 0.3
	87.7 (20)	82.8 (14.9)	81.2 (14.8)	NS
mean DAP (mmHg)	65.2 ± 10.7	63.2 ± 8.5	63.6 ± 10.9	*p* = 0.7
	63.4 (13.3)	63.7 (9.7)	63.7 (11)	NS
mean HR (beats/min)	61.1 ± 9.1	61 ± 7.7	60.9 ± 8.7	*p* = 1
	61.5 (11.7)	60.2 (11.2)	60.3 (12.4)	NS
mean SPI	36.6 ± 13.6	34 ± 10.5	35.2 ± 10.4	*p* = 0.8
	32.6 (17)	32 (13.3)	31.7 (13.7)	NS
mean SE	43.2 ± 10.6	40.4 ± 6.9	41.5 ± 7.1	*p* = 0.8
	41.3 (8.8)	41 (7.6)	40.6	NS
Stage 4—emergence from GA				
mean SAP (mmHg)	132.6 ± 26.5	124.4 ± 19.1	139 ± 27.3	M vs. P,
	127.3 (42)	124.3 (26)	138 (44)	*p* = 0.03
mean MAP (mmHg)	98.5 ± 17.6	93.5 ± 13.7	102.6 ± 16.8	M vs. P,
	97.8 (29.7)	92.5 (15.7)	102 (25.8)	*p* = 0.03
mean DAP (mmHg)	72.9 ± 12.3	70 ± 10.7	75.4 ± 11.4	*p* = 0.5
	72.8 (14.8)	69.5 (11.5)	74 (14)	NS
mean HR (beats/min)	61.2 ± 10.2	57.9 ± 6.8	61.1 ± 10	*p* = 0.2
	59.2 (12.7)	56.7 (11.5)	59.5 (11.6)	NS
mean SPI	49 ± 15	50.7 ± 14.1	53.7 ± 14.4	*p* = 0.3
	47.8 (22.6)	50.7 (22.9)	53.8 (22.6)	NS
Stage 5—PACU				
mean SAP (mmHg)	149.2 ± 18.6	147.6 ± 16.2	145.6 ± 18	*p* = 0.6
	150.3 (29.8)	147.4 (19.5)	142.3 (23.7)	NS
mean MAP (mmHg)	107.1 ± 10.2	104.4 ± 13.3	102.4 ± 14.5	*p* = 0.2
	106.3 (14)	104.8 (16.7)	101.3 (15.1)	NS
mean DAP (mmHg)	76.4 ± 10.1	77.7 ± 9.3	77.7 ± 10.7	*p* = 0.8
	76.8 (13.4)	75.7 (10.6)	78 (14)	NS
mean HR (beats/min)	69.3 ± 11.1	71.3 ± 11.1	68.5 ± 9.8	*p* = 0.6
	69.2 (13.7)	69.6 (10.6)	67 (13.4)	NS
mean SPI	55.9 ± 13.8	56.9 ± 16.3	51.4 ± 14.5	*p* = 0.2
	57.1 (23.1)	57.8 (26.1)	50.2 (23.5)	NS

PM—paracetamol/metamizole group; M—metamizole group; P—paracetamol group; SAP—systolic arterial pressure; MAP—mean arterial pressure; DAP—diastolic arterial pressure; HR—heart rate; SPI—surgical pleth index; VRS—vitreoretinal surgery; GA—general anesthesia; PACU—post-anesthesia care unit; Sd—standard deviation; Me—median; IQR—interquartile range; NS—not significant.

**Table 6:** Comparison of hemodynamic fluctuations of monitored patients’ parameters at the same stage between studied groups.

Parameter X ± Sd Me (IQR)	PM Group	M Group	P Group	*p*-Value
	N = 52 (34%)	N = 50 (32.7%)	N = 51 (33.3%)	
Stage 2—between induction and start of VRS				
max SAP (mmHg)	132.9 ± 31.8	134.6 ± 29.3	139.6 ± 27.7	*p* = 0.2 NS
	130 (39)	140 (40)	144 (44)	
max MAP (mmHg)	96.9 ± 19.9	101.2 ± 17.1	101.7 ± 17.8	*p* = 0.1 NS
	94.5 (23.5)	104 (24)	103 (25)	
max DAP (mmHg)	72.2 ± 14	77.5 ± 13.5	75.3 ± 12.8	*p* = 0.1 NS
	72.5 (17)	78.5 (23)	77 (19)	
max HR (beats/min)	71.9 ± 12.1	74.6 ± 11.7	74.5 ± 11.3	*p* = 0.3 NS
	70.5 (13.5)	75.5 (19)	74 (16)	
max SPI	40.6 ± 18.1	43.7 ± 13.5	44.2 ± 15.2	*p* = 0.2 NS
	39.5 (20)	42.5 (19)	43 (21)	
max SE	48.1 ± 11.1	49.5 ± 11	47.3 ± 10.4	*p* = 0.5 NS
	49 (13)	49.5 (16)	46 (17)	
min SAP (mmHg)	117.6 ± 31.3	116.7 ± 27.6	123.7 ± 28.9	*p* = 0.4 NS
	112 (43.5)	116 (36)	126 (47)	
min MAP (mmHg)	86.6 ± 20.2	89.4 ± 16.7	91.3 ± 19.2	*p* = 0.4 NS
	84.5 (27)	89 (24)	92 (29)	
min DAP (mmHg)	64.3 ± 14.6	68.7 ± 13.2	68.3 ± 13.6	*p* = 0.2 NS
	63.5 (18.5)	67 (24)	67 (22)	
min HR (beats/min)	63.8 ± 10.1	65.9 ± 10.2	64.9 ± 9.6	*p* = 0.5 NS
	61.5 (15)	67 (17)	64 (16)	
min SPI	27.3 ± 14.2	28.1 ± 12.5	29.3 ± 12.6	*p* = 0.4 NS
	23 (15)	25.5 (16)	25 (13)	
min SE	33.9 ± 10.5	33.6 ± 10	33.1 ± 10.4	*p* = 0.9 NS
	31 (17)	34 (8.7)	33 (13)	
Stage 3—VRS				
max SAP (mmHg)	138.2 ± 29.4	135.7 ± 30.1	137.8 ± 28.2	*p* = 0.9 NS
	128.5 (48.5)	132 (31)	134 (38)	
max MAP (mmHg)	101.7 ± 17.2	101.6 ± 19.3	103 ± 19	*p* = 0.9 NS
	100 (31.5)	98.5 (26)	99 (32)	
max DAP (mmHg)	76.6 ± 13	78.7 ± 14.9	76.6 ± 13	*p* = 0.8 NS
	75.5 (19)	78.5 (21)	75 (23)	
max HR (beats/min)	69.8 ± 11.6	71.7 ± 10.8	69.9 ± 10.8	*p* = 0.6 NS
	67.5 (16.5)	71.5 (17)	68 (19)	
max SPI	55.3 ± 15.4	56.2 ± 12.8	53.1 ± 11.7	*p* = 0.4 NS
	53.5 (25.5)	56 (19)	55 (16)	
max SE	53.7 ± 13.9	51.5 ± 9.1	54.4 ± 7.5	*p* = 0.1 NS
	50 (9)	51 (12)	56.5 (12)	
min SAP (mmHg)	103.5 ± 24.1	88 ± 13.9	93.8 ± 22.6	PM vs. M, *p* = 0.005
	96.5 (38.5)	87.5 (20)	87 (28)	
min MAP (mmHg)	77.2 ± 15.9	67.5 ± 10.7	70.7 ± 16	PM vs. M, *p* = 0.01
	73 (26.5)	66 (14)	66 (16)	
min DAP (mmHg)	57.7 ± 11.8	51.7 ± 9.1	53.2 ± 10.7	*p* = 0.05
	57 (18.5)	52 (13)	49 (15)	
min HR (beats/min)	56.3 ± 8.9	54.7 ± 7.9	55.3 ± 8.6	*p* = 0.7 NS
	56 (12)	53 (12)	54 (11)	
min SPI	24.4 ± 13.6	21 ± 7.8	23.5 ± 9.2	*p* = 0.5 NS
	20 (15)	21 (11)	22 (11)	
min SE	36.3 ± 12.3	32 ± 8.5	31.7 ± 6.9	*p* = 0.07 NS
	35 (12.5)	33 (10)	32 (7)	
Stage 4—emergence from GA				
max SAP (mmHg)	144.3 ± 33.8	132.2 ± 22.5	144.9 ± 28.1	*p* = 0.1 NS
	139.5 (52)	131 (29)	146 (51)	
max MAP (mmHg)	104.3 ± 20	99.4 ± 16.2	106.7 ± 17.5	*p* = 0.1 NS
	103.5 (36)	99 (20)	107 (28)	
max DAP (mmHg)	76.3 ± 13.9	73.5 ± 12.1	78.2 ± 11.7	*p* = 0.2 NS
	76 (16)	73 (16)	78 (12.5)	
max HR (beats/min)	69.4 ± 12.7	63.2 ± 8.6	67.4 ± 12	PM vs. M, *p* = 0.04
	67 (18)	61.5 (13)	65 (19)	
max SPI	63.5 ± 16.1	64.1 ± 12.7	64.1 ± 14.3	*p* = 1 NS
	65 (23)	64.5 (17)	64 (20)	
min SAP (mmHg)	122.8 ± 23.1	118.5 ± 19.6	133.3 ± 29	M vs. P, *p* = 0.02
	121 (37)	120 (28)	130 (40)	
min MAP (mmHg)	91 ± 15.1	90.4 ± 14.5	98.3 ± 18	*p* = 0.05 NS
	88.5 (23)	90 (20)	98 (28)	
min DAP (mmHg)	66.6 ± 12.6	66.9 ± 11	72.5 ± 12.3	PM vs. P, *p* = 0.04; M vs. P, *p* = 0.03
	66 (15)	66 (14)	72 (16)	
min HR (beats/min)	57.5 ± 9.3	55.6 ± 6.7	57.4 ± 10.1	*p* = 0.8 NS
	55.5 (12.5)	56 (10)	56 (12)	
min SPI	38.3 ± 16.5	38.7 ± 15.2	43.5 ± 17.1	*p* = 0.2 NS
	36.5 (29)	35.5 (25)	42 (28)	
Stage 5—PACU				
max SAP (mmHg)	156.7 ± 20.8	155.2 ± 19.3	153.1 ± 19.1	*p* = 0.8 NS
	154 (33)	154 (18)	153 (26)	
max MAP (mmHg)	113.1 ± 10.9	111.2 ± 15.5	108.1 ± 14.5	*p* = 0.2 NS
	112 (16)	111.5 (19)	107 (17)	
max DAP (mmHg)	81.6 ± 10.5	100.3 ± 110.3	83.1 ± 12.1	*p* = 0.8 NS
	81 (15)	82 (14)	82 (12)	
max HR (beats/min)	75.3 ± 12.7	76.1 ± 12.1	73.3 ± 10.7	*p* = 0.7 NS
	73 (17)	74 (12)	73.6 (17)	
max SPI	69 ± 11.3	66.5 ± 16.5	60.4 ± 15.1	PM vs. P, *p* = 0.03
	71 (16)	69 (27)	58 (26)	
min SAP (mmHg)	142.1 ± 18.3	139.4 ± 13.8	139.5 ± 18.1	*p* = 0.6 NS
	139 (26)	140 (23)	135 (24)	
min MAP (mmHg)	101.7 ± 10.5	100 ± 12.8	97.8 ± 15.7	*p* = 0.5 NS
	102 (17)	102.5 (17)	98 (18)	
min DAP (mmHg)	71.1 ± 10.6	72.7 ± 9.5	73.5 ± 11.1	*p* = 0.6 NS
	73 (16)	73 (15)	73 (18)	
min HR (beats/min)	63.6 ± 10.8	66 ± 11.1	64.6 ± 9	*p* = 0.4 NS
	62 (10)	65 (13)	64 (13)	
min SPI	44 ± 17.7	48.1 ± 16.2	43.5 ± 14.6	*p* = 0.4 NS
	45 (30)	49 (28)	43 (23)	

PM—paracetamol/metamizole group; M—metamizole group; P—paracetamol group; SAP—systolic arterial pressure; MAP—mean arterial pressure; DAP—diastolic arterial pressure; HR—heart rate; SPI—surgical pleth index; VRS—vitreoretinal surgery; GA—general anesthesia; PACU—post-anesthesia care unit; Sd—standard deviation; Me—median; IQR—interquartile range; NS—not significant.

## Data Availability

The data used to support the findings of this study are included within the article.
